# Letter from Prof. John C. Dalton, Jr.

**Published:** 1852-09

**Authors:** Jno. C. Dalton

**Affiliations:** Paris


／# BUFFALO MEDICAL JOURNAL

AND

### MONTHLY REVIEW.

VOL. 8. SEPTEMBER, 1852. NO. 4.

### ORIGINAL COMMUNICATIONS.

### ART. I. — *Letter from Prof. John C. Dalton, Jr.*

### PHYSIOLOGICAL RESEARCHES OF M. BERNARD.

PARIS, June 21, 1852.

DEAR DOCTOR, — According to M. Bernard's view of the action of the gastric fluid and the changes which the food undergoes in the stomach, it will readily be understood that the "chyme" will vary considerably in composition with the nature of the alimentary materials. It has been formerly supposed that the chyme was a homogenous fluid, resulting from the indiscriminate solution of *all* the digestible substances which had been taken into the stomach. In reality it is not so; — since it is only the albuminoid matters which are digested in the stomach, and the remaining alimentary principles pass into the intestine in the same, or nearly the same condition in which they were swallowed. The chyme, then, is a mixture of digested albuminoid matters, and undigested, oily, starchy, and saccharine substances. It remains to be seen what becomes of these last. Our method of ascertaining where and by what agents these matters are digested is to follow them downward in the intestine and discover at what point of the alimentary canal the oil, or the starch, loses its natural physical and chemical properties and becomes absorbable. Oily matters, for example, enter the duodenum unchanged; — but at a certain distance from the pylorus they suffer an alteration, are digested, in fact, and are no longer to be recognized by their ordinary characters. This change commences in many animals immediately below the opening of the biliary and pancreatic ducts, and as the bile appeared to be the most abundant and important intestinal fluid, the digestion of fatty substances has been attributed by some experimenters to this secretion. In some animals, however, the ducts are separated by a considerable distance; and in all these instances it is the biliary duct which comes first,— the pancreatic duct afterward. In the rabbit, for example, the biliary duct opens at the upper part of the duodenum, while the pancreatic joins the intestine some eighteen inches farther down. In all cases where the ducts are so separated the fat can still be recognized in the intestine after it has passed the opening of the biliary duct, and disappears only when it has been subjected to the action of the pancreatic fluid. The digestion of oily matters, in other words, always corresponds, in place, to the opening of the pancreatic duct. It occurs high up in the intestine when the duct is situated high, and lower down when the duct opens lower down. There is, then, the most complete proof that can be drawn from comparative anatomy, that it is the pancreatic fluid that accomplishes the digestion of oily substances.

But a still more convincing proof is obtained by the method which M. Bernard has already followed with the saliva and the gastric juice, viz., by obtaining the fresh secretion from a living animal and trying, by direct experiment, its action on the alimentary principles. For this purpose he takes a dog in whom the processes of digestion are actively going on, makes a short incision into the abdomen a little to the right of the median line, finds the pancreatic duct *by feeling*, introduces a slender silver canula and allows the secretion to drain away into a small India rubber reservoir, until enough has been accumulated for experiment. This requires only a short time if digestion is going on when the operation is done. He then takes fresh bile, saliva (the different varieties) and gastric fluid, obtained in the same manner. Olive oil shaken up with these different fluids in test tubes is only mechanically mixed with them; but when it is poured into a test tube containing fresh pancreatic fluid, it is immediately *emulsioned* in the most complete manner; — and the fluid, which was transparent and limpid like water, becomes at once white and opaque as milk. If the emulsion be exposed for some time to the air at a temperature of 40° cent. it suffers a further change and from alkaline becomes acid, in consequence of the fat being decomposed into glycerine and a free fatty acid. This last change, however, is entirely artificial and does not take place in natural digestion. In the intestine the oil is simply *emulsioned*, and still retains its peculiar *chemical* characters. It is, therefore, absorbed as oil, but in a state of minute subdivision.

When the fresh pancreatic secretion is obtained from a living animal in the manner indicated above, it is a clear, watery fluid, with a distinct alkaline reaction. It has the following composition:

| | |
|---|---|
| Water, - | 91.28 |
| Organic Matter, | 0.44 |
| Ashes, - | 8.28 |
| | 100.00 |

Free Soda,
Chloride of Sodium,
" " Potassium,
Lactate of Lime,
Alkaline Carbonates,
Phosphate of Lime.

To which of these ingredients does the pancreatic fluid owe its peculiar property of emulsioning fat? It is not the free soda or the alkaline carbonates, since the saliva and the bile are equally alkaline but have no similar effect on oils. The contents of the intestines also are in many animals, constantly acid, and would therefore effectually prevent any action that depended on the alkaline qualities of the secretion. The pancreatic fluid, like the gastric juice, owes its digestive properties to the organic matter which it holds in solution. This organic matter, like that of the gastric juice, is precipitable by alcohol; and the precipitate, drained, washed, and redissolved in water, retains all its original properties. Its solution has the property of emulsioning oily substances in the same manner as the natural pancreatic fluid.

The active principle of the pancreatic fluid has some points of resemblance with albumen, since it is precipitated both by heat and alcohol. It is not albumen, however, since neither the white of an egg nor the albumen of the blood have any similar action on oils. It differs from albumen, also, in some chemical characters; as its alcoholic precipitate is easily soluble again in water, while albumen once precipitated cannot be again dissolved.

It resembles casein, also, in some respects. It is precipitated, for example, like casein, by sulphate of magnesia in excess. As the casein in milk apparently seems to hold its oily part in emulsion, so the pancreatic fluid in the intestines exerts a similar action on the oleaginous ingredients of the food; so that there is, in reality, considerable resemblance in the physiological properties of the two substances. They are not, however, identical, since the pancreatic fluid is coagulated by heat, which has no effect on a solution of casein. Bernard finds that fresh pancreatic fluid constantly coagulates by heat. Other experimenters, particularly in Germany, have stated the contrary, and maintained that the secretion was unaffected by heat. This difference in the result of the experiments Bernard explains by the fact that the pancreatic secretion becomes altered very soon after the operation of making an artificial fistula. Even in dogs, who bear these operations on the abdomen with greater impunity than most animals, the partial peritonitis, which is soon established about the wound, vitiates the secretion of the pancreas to such a degree, that it will no longer coagulate by heat, nor exert its proper action on oily substances. For purposes of experiment, it is always necessary to take the *first fluid secreted* after the performance of the operation.

The active principle of the pancreatic fluid must, then, be considered as a peculiar organic matter, which gives to the secretion the power of emulsioning oily substances.

There is still another class of alimentary principles, namely, the amylaceous, which acquire to be modified by the action of the intestinal fluids. Starch is no more absorbable in its natural condition than fat, and, to become absorbable, it is transformed first into dextrine and next into sugar; and it is finally absorbed into the circulation under the form of sugar. There is one thing, however, remarkable about the digestion of starchy substances. While the digestion of both the other orders of alimentary principles, albuminoid and oleagious, is strictly *localized*, so to speak, i. e., is performed in particular parts of the alimentary canal, and by means of special secretions, the digestion of starchy substances is not so; — but takes place indiscriminately throughout the whole length of the digestive tube, below the stomach. *All* the intestinal fluids have, more or less, the property of converting starch into sugar. Simple contact with any part of the intestinal mucous membrane is alone sufficient to effect the change.

There are, then, three different digestions, so to speak, carried on in the alimentary canal; a different process being required for each of the three principal orders of alimentary substances; and at the same time there are three different products resulting from their modification. First, the albuminoid matters are dissolved by the gastric fluid in the stomach and converted into "albuminose;" a substance which is not coagulated by heat or the strong acids, but only by some metallic salts. Albumen, fibrin, and casein, are all three converted by stomach digestion into this secondary principle. Second, fatty substances are converted into an oily emulsion by the pancreatic fluid in the duodenum; and thirdly, starch is transformed into sugar by the action of the intestinal fluids generally.

It will be seen that no account has yet been given of the nature and action of the bile; a secretion which seems particularly difficult of study, notwithstanding its great abundance and the ease with which it may be obtained for purposes of experiment. M. Bernard's explanation of its physiological properties will not, probably, appear by any means so clear and satisfactory as that which he gives of the other digestive fluids. One cannot help suspecting indeed, that he is not entirely satisfied with his own ideas on this point. Such as they are, however, you shall have them, and form your own opinion as to their merits.

It has already been said that the bile has the property of converting starch into sugar. But this property is one which it possesses in common with all the other intestinal fluids and cannot be considered as at all characteristic. The bile, in fact, has by itself no special action on any of the alimentary principles. Neither oleaginous nor albuminoid matters, in their natural condition suffer any change by being placed in contact with it. But if albuminoid matters, which have already passed through the stomach be mixed with bile, an immediate action becomes evident. In all animals the opening of the biliary duct is situated at a very short distance from the pylorus; so that the food, on passing out of the stomach, comes immediately in contact with the biliary secretion. The effect of this contact is to produce a copious yellow precipitate. Matters which were held in solution by the gastric fluid are thrown down by the bile. In other words, the chemical actions which had been going on in the stomach are arrested as soon as the food enters the duodenum. At this part of the alimentary canal, a new set of actions is about to commence; and in order that they may be properly accomplished it is necessary that those which have preceded them should be checked. For there is an essential difference, a kind of opposition, between the changes which the food undergoes in the stomach, and those which are to take place in the intestine. The processes of stomach digestion are essentially antiseptic. They are analogous to those produced by the prolonged action of fire. Fibrin, for example, is transformed into albuminose. Fat which is not chemically changed, is simply *melted*. Starch in the stomach merely swells up, and becomes hydrated, exactly as it does by boiling in water. On all the alimentary matters the effect of stomach digestion is analogous to a kind of cooking; and is entirely *anti-putrefactive.* The gastric fluid is itself anti-putrefactive, and very little liable to change. It may be preserved for an indefinite length of time without losing its digestive properties.

On the other hand, the pancreatic fluid is extremely liable to putrefaction and changes very rapidly by exposure; so that a very short time after it has been secreted it can no longer be used for purposes of experiment. Since there is this opposition between the actions of the gastric and pancreatic fluids, they would necessarily interfere with each other, were there not some secretion intermediate between the two, which should neutralize the action of the gastric juice before the contents of the stomach come to be mingled with the pancreatic fluid. The bile is such a secretion. It immediately destroys the gastric fluid, and arrests its action; and, in fact, it is found by direct experiment, that if bile be injected into the stomach of a living animal, it effectually stops, for a time, the digestive process.

Another effect of the presence of bile in the intestine, seems to be to regulate the chemical changes which go on there, in such a way that the products of these changes are not the same that they would be out of the body. The decomposition of azotized organic matter, for example, out of the body, always gives rise to ammoniacal products. On the other hand, the substances resulting from the decomposition of non-azotized matters are always acid. Starch is transformed first into dextrine and sugar, and a continuation of the process produces lactic acid. Fats are decomposed into glycerin and a free fatty acid. In the intestine, however, exactly the contrary is the case. The internal surface of the intestine of carnivorous animals has always an acid, that of herbivorous animals an alkaline reaction. The azotized matters give rise to acid products, and the non-azotized to alkaline. This modification of the chemical changes, as they take place in the intestine, is referred to an influence exerted by the presence of the bile.

Such is M. Bernard's account of the character and functions of the bile. The secretion is so evidently one of a very complicated character, that perhaps it is not surprising that we have not yet entirely mastered its physiological history. Unlike other secretions, a large portion of it, after being once poured out by the excretory duct is reabsorbed, during its passage down the intestine. In the rabbit, for example, it is estimated that a quantity of bile is secreted daily amounting to one-eighth or one-tenth the weight of the whole body. But four-fifths of this quantity are reabsorbed before it reaches the end of the intestine, and it is only a small portion, consisting mainly of the bitter principle and coloring matter which is finally rejected with the refuse parts of the food. The liver is not only a secretory and an excretory organ, but is destined at the same time, to accomplish certain other processes in the preparation of the blood which are still more obscure and complicated; as will be seen from what follows on the absorption of the digested portions of the food.

The three alimentary substances, which have been subjected to different digestive processes in the stomach and intestine, and which have respectively been converted into albuminose, sugar and an oily emulsion, are afterward absorbed into the circulation. But they are not all absorbed by the same vessels. There are two different routes which these substances follow in leaving the cavity of the intestinal canals: 1st, the portal vein; 2d, the lacteals. *All the albuminoid and amylaceous substances pass by the portal vein; all the fatty matters are taken up by the lacteals.* The chyle, which was formerly supposed to contain all the products of digestion, in reality contains only one class of them, the oleaginous. The two other classes are absorbed by another system of vessels. The anatomy of the portal vein on the one hand, and of the lacteals on the other, make it evident that all the alimentary materials, after absorption, and before entering the general circulation, are compelled to pass through certain preparatory organs. The oleaginous matters, entering the subclavian vein by the thoracic duct, are taken directly to the lungs. The albuminoid and amylaceous substances, taken up by the portal system, must pass through the liver before they are mingled with the rest of the blood in circulation. In these organs, the substances which have been absorbed, are destined to undergo a further modification before they can be used for purposes of assimilation. Even albumen is not assimilable until it has passed through the hepatic circulation. If pure albumen be injected into the *jugular* vein of an animal, it is not assimilated, but is excreted in the urine, as albumen. But if it be injected into the *portal* vein, it passes through the liver, becomes assimilable, and no longer appears in the urine. Cane sugar, absorbed by the portal system is converted in the liver into grape sugar. Oleaginous matters suffer some analogous change in the lungs, by which they are rendered fit to be used in the processes of nutrition. For these reasons it appears at least doubtful whether it be possible to support life to any extent by means of "nutritive baths," which have sometimes been used for the purpose. Nutritive enemata are undoubtedly useful, since the albuminoid matters are taked up by the portal system and carried to the liver. But when absorbed by the vessels of the skin, they are not yet fit for assimilation, and must therefore be excreted in the same manner as when injected into the jugular vein. The digestive processes, therefore, or that by which the elements of the food are prepared for conversion into blood, far from being a simple process, performed in the stomach alone by the gastric juice' is not even completed in the interior of the alimentary canal. But the nutritive matters, after being rendered absorbable have still other changes to undergo in the lungs and liver by which they are made assimilable; and these must necessarily be considered as essential parts of the process of digestion.

A few days ago, I had the pleasure of seeing, in M. Bernard's laboratory, two experiments which I had heard of before, but which it is difficult to believe in thoroughly, without the evidence of one's own senses. The first was a demonstration of the manufacture of sugar in the liver; the second, the production of diabetes mellitus in a rabbit, by wounding the posterior part of the medulla oblongata. Both experiments were completely successful.

M. Bernard maintains that one of the constant and normal functions of the liver is the production of sugar. In all animals, dead suddenly while in good health, in the human subject under similar circumstances, in executed criminals, &c., the blood of the liver and of the hepatic vein, and the substance of the liver itself is found to contain a very appreciable quantity of sugar (glucose); and this, when no sugar or starch has been taken for food, and when it cannot be discovered in the contents of the intestines nor in the blood of the portal vein below the liver. Any serious indisposition checks this production of sugar, in the same way as it checks the secretion of gastric juice, of cutaneous perspiration, &c. But in a state of health it is a constant phenomenon. The sugar thus produced by the liver is destroyed in the lungs; consequently it is not found in the general circulation, nor in any other organ in the body than the liver.

To prove this fact M. Bernard took a young dog that had been fed all the morning on animal food, and killed him instantaneously by destroying the medulla oblongata with a kind of "garrote." The abdomen was immediately opened, and a ligature placed on the portal vein just below the liver, another on the hepatic vein just above, and a quantity of blood taken from each of these two situations to be tested. Each portion was subjected to the same processes of coagulation, decolorizing, &c. — and afterward treated by the same reagents; tartrate of potass and copper, with heat. The blood from the portal vein, which had not yet passed through the liver showed no alteration whatever; in the other specimen a copious precipitate of the suboxide of copper took place, as abundant as is often seen in cases of diabetes mellitus. The substance of the liver, brayed in a mortar and extracted with water, showed the same reaction, while the substance of the lungs, treated in a similar manner, showed nothing of the kind. The fermentation test was also applied, but was altogether superfluous, as the results of the first were completely satisfactory.

The second experiment is as follows: a rabbit is taken and the bladder emptied by compressing the hypogastrium. The urine is tested for sugar, and, as might be expected, shows no trace of it. A small steel instrument is then passed through the posterior part of the skull, into the substance of the medulla oblongata, and is immediately withdrawn. The instrument has a transverse cutting edge, like a chisel, a little over one line in length. From the middle of this edge a fine steel point runs out in the axis of the shaft for about two lines. This point is to prevent the cutting edge from passing through the medulla, and wounding its anterior fibres, which would destroy the animal. It is the posterior portion of the medulla alone which is to be wounded by the cutting edge. The instrument is then passed forward in the median line until the steel point rests upon the basilar process of the occipital bone, when it is immediately withdrawn. If the puncture has been made accurately in the median line, the animal makes no struggle during the operation, and appears simply feeble and exhausted afterward. He soon attempts, indeed, to use his limbs, and in a few days is generally quite recovered. If the instrument has divided considerably to either side, the animal presents the singular phenomenon of constantly turning toward the wounded side whenever he attempts to move. In the course of two hours after the operation, the urine tested by the tartrate of potass and copper, shows an abundance of grape sugar in solution; and the blood taken from the jugular vein also contains a considerable quantity. This state of artificial diabetes continues, in the rabbit, from thirty-six to forty-eight hours. At the end of that time the sugar disappears from the blood and secretions, and the animal returns to its natural condition, after which the state of diabetes may be again produced by a fresh puncture. Indeed the experiment, if carefully performed, may be repeated several times on the same animal. The explanation of this singular phenomenon is not altogether easy. It is considered by M. Bernard as illustrating the connection between the cerebro-spinal and sympathetic nervous systems. But however it may be interpreted, the fact itself is incontestible.

Yours truly,

JNO. C. DALTON, Jr.

